# Myricetin inhibits pseudorabies virus infection through direct inactivation and activating host antiviral defense

**DOI:** 10.3389/fmicb.2022.985108

**Published:** 2022-09-15

**Authors:** Huaiyue Hu, Zhiqiang Hu, Yingying Zhang, Hongping Wan, Zhongqiong Yin, Lixia Li, Xiaoxia Liang, Xinghong Zhao, Lizi Yin, Gang Ye, Yuan-Feng Zou, Huaqiao Tang, Renyong Jia, Yaqin Chen, Hao Zhou, Xu Song

**Affiliations:** ^1^Natural Medicine Research Center, College of Veterinary Medicine, Sichuan Agricultural University, Chengdu, China; ^2^Shandong New Hope Liuhe Agriculture and Animal Husbandry Technology Co., Ltd., Dezhou, China; ^3^College of Animal Science and Technology, Sichuan Agricultural University, Chengdu, China; ^4^College of Medical Technology, Chengdu University of Traditional Chinese Medicine, Chengdu, China; ^5^Department of Microbiology, NYU Grossman School of Medicine, New York, NY, United States

**Keywords:** myricetin, dietary flavonoids, herpesvirus, pseudorabies virus, antiviral activity

## Abstract

Myricetin, a polyhydroxyflavone compound, is one of the main ingredients of various human foods and therefore also known as dietary flavonoids. Due to the continuous emergence of resistant strains of herpesviruses, novel control measures are required. In the present study, myricetin exhibited potent antiviral activity against pseudorabies virus (PRV), a model organism of herpesvirus. The suppression rate could reach up to 96.4% at a concentration of 500 μM in cells, and the 50% inhibitory concentration (IC_50_) was 42.69 μM. Moreover, the inhibitory activity was not attenuated by the increased amount of infective dose, and a significant reduction of intracellular PRV virions was observed by indirect immunofluorescence. A mode of action study indicated that myricetin could directly inactivate the virus *in vitro*, leading to inhibition of viral adsorption, penetration and replication in cells. In addition to direct killing effect, myricetin could also activate host antiviral defense through regulation of apoptosis-related gene expressions (Bcl-2, Bcl-xl, Bax), NF-κB and MAPK signaling pathways and cytokine gene expressions (IL-1α, IL-1β, IL-6, c-Jun, STAT1, c-Fos, and c-Myc). In PRV-infected mouse model, myricetin could enhance the survival rate by 40% at 5 days post infection, and viral loads in kidney, liver, lung, spleen, and brain were significantly decreased. The pathological changes caused by PRV infection were improved by myricetin treatment. The gene expressions of inflammatory factors (MCP-1, G-CSF, IL-1α, IL-1β, and IL-6) and apoptotic factors (Bcl-xl, Bcl-2, and Bax) were regulated by myricetin in PRV-infected mice. The present findings suggest that myricetin can effectively inhibit PRV infection and become a candidate for development of new anti-herpesvirus drugs.

## Introduction

Myricetin, a polyhydroxyflavoid compound, was first found in bayberry bark in the late 18th century (Perkin and Hummel, 1896), and then successively isolated in other plants, such as Myricaceae, Vitaceae, Fabaceae, Ericaceae, and Euphorbiaceae (Liu and Yu, 2014). It is also one of the main ingredients of various human’s foods, such as fruits, vegetables, fruit wine, tea, and honey, thus also known as dietary flavonoids (Deepak et al., 2016). The content of myricetin varies in different plants. It is well known that large amount of myricetin is present in the red bayberry trees which up to 10.53 mg/g in the red bayberry leaves (Yuan-Dan et al., 2015), but in bayberry core only 0.05 mg/g (Zou, 1995). The content of myricetin in fresh strawberries is only 75.7 μg/g (Gema and Maria, 2016). *Xanthoceras Sorbifolia Bunge* is a kind of Tibetan medicine, mostly grown in Inner Mongolia, and has the content of myricetin about 0.8 mg/g (Yang et al., 2014). Vine tea is known as the “king of flavonoids” and contains 44% of total flavonoids, in which myricetin content was 6.73 mg/g (Chen et al., 2015). Zhang et al. (2021) compared 22 different sources of Xinyang Maojian tea and found that the content of myricetin reach up to 0.370 mg/g. As a natural flavonoid, myricetin has been developed as medicines, foods, health care products and cosmetics. Previous studies have shown that myricetin has a strong antioxidant activity to scavenge a series of free radicals and ions such as DPPH, NO (Rostoka et al., 2010), ROS (Di et al., 2011). It can protect the organism from oxidative damage, and has both antioxygen and promoting oxidation which depending on ascorbic acid and Fe3^+^ involvement (Chobot and Hadacek, 2011; Duthie and Morrice, 2012). Myricetin has a wide range of anti-cancer effects, which is not only cytotoxic to various cancer cells such as liver, lung, and skin, but also inhibits the enzymatic activity associated with the development of cancer (Ci et al., 2018; Li et al., 2019; Xie et al., 2019). The anti-inflammatory activity of myricetin has been demonstrated in many trials through regulation of various signaling pathways (Hou et al., 2018; Kan et al., 2019). Myricetin could inhibit the growth of both bacteria and viruses (Cai and Wu, 1996; Naz et al., 2007). It was reported that myricetin could target the viral gD protein and downregulate the cellular EGFR/PI3K/Akt pathway to inhibit HSV replication (Li et al., 2020). Myricetin could inhibit HIV infection through suppression of SEVI fibers formation (Ren et al., 2018) and a direct killing effect (Pasetto et al., 2014). It could inhibit the activation of reverse transcriptase of murine leukemia virus (RLV) and HIV (Ono et al., 2010) and the activation of SARS-CoV helicase protein (nsP13; Yu et al., 2012). In addition, myricetin also has anti-allergy, antiacid, antihypertensive, and analgesic activities.

Herpesvirus, a class of viruses with double-stranded DNA, mainly invade skin, mucosal and neural tissues (Wildy, 1985), and then cause latent infection which is one of the major features of herpesvirus (Cohen, 2020). It can infect a wide range of hosts including amphibians, mammals and primates, and also involves in the development of certain cancers and causes complications of multiple diseases (Rafferty and Keen, 1973; Burrows, 1977; Boštíková et al., 2014). Pseudorabies virus (PRV), also known as swine herpes virus type I, belongs to the α subfamily of the *Herpesviridae* family. It is the pathogen that causes various mammalian Aujeszky’s disease (Wittmann and Rziha, 1989). Viruses in the α subfamily were found to be highly homologous, so PRV is often served as a model virus for studying α-herpesviruses (Karlin et al., 1994). Because of its high neurophilicity, PRV is often used as the “live” tracer of neuropathways (Sun et al., 2019). Although previous studies found that there was no cross-reactive between antibodies to PRV and HSV (Robbins et al., 1987), cases of humans infected with PRV have occurred since 1914. The patients developed symptoms associated with the CNS and tested positive for antibody specificity at 5–15 months of onset (Mravak et al., 1987; Skinner et al., 2001). Recent reports showed that some patients suffered from endophthalmitis and encephalitis caused by the variant PRV strain (Ai et al., 2018). Thus, it appears that PRV is most likely to vary into the neglected zoonosis (Wong et al., 2019). Although many trials have been conducted, vaccines against the human herpes virus are still not approved (Rajčáni et al., 2018). The approved drugs, including acyclovir, valacyclovir, and penciclovir are a class of nucleoside analogues that strongly inhibit DNA polymerase, which can effectively prevent and treat primary infection of herpes virus, but cannot protect the body from the damage caused by secondary infection (Sawleshwarkar and Dwyer, 2015). Studies have found that some compounds extracted from natural plants have good anti-herpesvirus activity, especially flavonoids (Ninfali et al., 2020). They can function at various stages of the viral infection cycle, providing new strategies for the treatment of herpesviruses. In this study, the antiviral activity of myricetin against PRV was evaluated in order to developing a new treatment for herpesvirus infection from dietary flavonoids.

## Materials and methods

### Reagents cells and virus

Myricetin (MB1893-2; with purity > 95%) was purchased from Dalian Meilun Biotechnology Co., Ltd. (Dalian, China). Pig kidney cells (PK-15) were preserved in the Natural Medicine Research Center Sichuan Agricultural University (Chengdu, China) and grown in high glucose medium (DMEM) containing 10% fetal bovine serum (PAN), penicillin (100 U/ml), and streptomycin (100 μg/ml) at 37°C 5%CO_2_. PRV (Ra strain) was purchased from China Veterinary Culture Collection Center (Beijing, China) and maintained at the Natural Medicine Research Center Sichuan Agricultural University (Chengdu, China) after propagated for five generations in PK-15 cells.

### Half cytotoxic and half inhibition concentrations assays

The cytotoxic effects of myricetin on PK-15 cells were determined by cell counting kit-8 assay (CCK-8; MA0218-T; Dalian Meilun Biotechnology Co., Ltd.) according to the manufacturer’s instructions. PK-15 monolayers grown in 96-well plates were incubated with DMEM containing 2% fetal bovine serum and 2-fold dilutions of myricetin for 48 h at 37°C. Then 10 μl CCK-8 solution was added to each well and incubated at 37°C for 30 min, followed by measurement of absorbance values at 450 nm in a microplate reader (Bio-Rad, United States). The 50% cytotoxic concentration (CC_50_) was calculated by the Reed-Muench method.

The antiviral activity of myricetin was evaluated by the cytopathic inhibition assay (Yang et al., 2016b). PK-15 cells grown in 96-well plates were infected with PRV (100 TCID_50_) at confluence of 80%–90% in the presence of different concentrations of myricetin. After infection for 1 h, the virus-containing culture medium was removed and different concentrations of myricetin diluted in culture medium were added again. When the infected cells without any treatment showed approximately 80% cytopathic effect (CPE), CCK-8 assays were performed as described above. The 50% inhibitory concentration (IC_50_) was calculated by the Reed-Muench method.

### Indirect immunofluorescence assay

The indirect immunofluorescence assay was conducted as previously described (Wang et al., 2017). PK-15 cells were infected with PRV (MOI = 0.1) in the presence of myricetin (500 μM), after adsorption for 1 h at 37°C, the virus-containing medium was replaced with a culture medium containing myricetin. After incubation for 24 h, the cells were washed with PBS and fixed with 4% paraformaldehyde for 15 min. After penetration with 0.2%TritonX-100 for 10 min, the cells were incubated with 5% BSA for 30 min at 37°C. After washing, cells were incubated consecutively with anti-PRV antibodies (ab3534, Abcam, 1:200 dilutions) and FITC Conjugated Goat Anti-rabbit IgG (H + L; BA1105; BOSTER, Wuhan, China). Then, the cell nucleus was stained with DAPI for 5 min before fluorescence microscope observation. Images were recorded using a Nikon fluorescence microscope (80i).

Anti-PRV activity of myricetin against different viral dose PK-15 cells was infected with PRV (MOI = 1, 0.1, and 0.01, respectively) in the presence of myricetin (500 μM), after adsorption for 1 h at 37°C, virus-containing medium was removed and culture medium containing myricetin was added. After incubation for 24 h, total DNA of cells was extracted to detect viral DNA copy by using DNAiso Reagent (D305; TaKaRa, China) according to the manufacturer’s instructions. For determination of viral gene copies, fluorescent quantitative PCR (FQ-PCR) was performed using a Bio-Rad CFX96 Connect^TM^ Real-Time PCR Detection System (CA, United States) according to the method described by Zhao et al. (2017).

### Mode of action assay

In order to elucidate which stage of viral infection was inhibited by myricetin, the mode of action assay was performed by the following five tests (Chen et al., 2021). (1) Pretreatment: cells were incubated with myricetin (500 μM) for 1 h at 37°C prior to PRV infection (100 TCID_50_). (2) Inactivation: PRV (10,000 TCID_50_) was incubated with myricetin (500 μM) at 37°C for 1 h followed by 100-fold dilution, and then the mixture was added to cells for infection. (3) Adsorption: cells were infected with PRV (100 TCID_50_) in the presence of myricetin (500 μM) at 4°C for 1 h, and then the cells were washed to remove unadsorbed viruses, followed by incubation at 37°C. (4) Penetration: PRV (100 TCID_50_) was allowed adsorption to pre-cooled cells for 1 h at 4°C, followed by incubation with myricetin (500 μM) at 37°C for 1 h. (5) Post-infection: cells were infected with PRV (100 TCID_50_) for 1 h at 37°C, followed by addition of myricetin (500 μM) after removed the unadsorbed virus. After incubation for 48 h, the viral DNA copy in each assay were measured by FQ-PCR method as described above.

### Virucidal activity

The method for detecting virucidal activity was described previously (Jones et al., 2020). The PRV at 10^7.3^ TCID_50_ (0.1 ml) was incubated with or without equal volume of myricetin (1,000 μM) at 37°C for 0, 5, 10, 15, 30, 45, and 60 min and then serially 10-fold diluted on PK-15 cells in 96-well plates. After incubation at 37°C for 48 h, the viral titer was detected by the Reed-Muench method.

### Effect of myricetin on the PRV growth curve

PK-15 cells seeded in 6-well plates were infected with PRV (MOI = 0.1), and after incubation for 1 h at 37°C, the medium was replaced by the myricetin-containing (500 μM) culture medium. Cells were collected to extract viral DNA at the following each time point: 2, 4, 6, 8, 12, 18, and 24 h post infection (hpi), respectively. The viral DNA copy were detected by FQ-PCR.

### Apoptosis

The effect of myricetin on PRV causing apoptosis in PK-15 cells was determined by Annexin V-FITC/PI double staining (Hu et al., 2019). PK-15 cells seeded in 6-well plates were infected with PRV (MOI = 0.1). After adsorption for 1 h, virus-containing medium was replaced by the myricetin-containing (500 μM) culture medium. The cells were incubated for 24 h, and then, Annexin V-FITC of binding solution (195 μl), AnnexinV-FITC (5 μl), and PI of staining solution (10 μl) were consecutively and gently added and mixed. After incubation from light at room temperature for 20 min, images were recorded using a Nikon fluorescence microscope (80i, Japan).

### Western blot assay

PK-15 cells were infected with or without PRV (MOI = 1), and after adsorption for 1 h virus-containing medium was replaced by the culture medium with or without myricetin (500 μM). Total proteins of cells were extracted with a commercial kit (BOSTER, Wuhan, China) at 4, 8, and 12 hpi, respectively. Then, the proteins were isolated by 10% sodium dodecyl sulfate-polyacrylamide gel electrophoresis (SDS-PAGE) and transferred to polyvinylidene difluoride (PVDF) membranes (Millipore, United States). The membrane was blocked in 5% (w/v) skim milk diluted with Tris-buffered saline containing 0.1%(v/v) Tween 20 (TBST) for 90 min at room temperature, followed by incubation with primary antibodies against P65 (CST, United States, 1:1,000), p-P65 (CST, United States, 1:1,000), P38 (CST, United States, 1:1,000), p-P38 (CST, United States, 1:1,000), ERK1/2 (CST, United States, 1:1,000), p-ERK1/2 (CST, United States, 1:1,000), and β-actin (Boster, Wuhan, 1:1,000) at 4°C overnight, respectively. The membranes were washed with TBST and incubated with horseradish peroxidase-conjugated secondary antibody (CST, United States, 1:5,000) at room temperature for 1.5 h. The proteins were visualized using enzymatic chemiluminescence (ECL) reagents (Bio-Rad). The expressions of total proteins were normalized according to the expression of β-actin and ratios of protein band intensities were obtained with ImageJ Software (Version 1.47; NIH, United States).

### Cytokines assay

Total RNA of PK-15 cells and organs was extracted by TRIzol reagent (Invitrogen, United States) according to the manufacturer’s protocol. Then, the total RNA was reverse-transcribed by Revert Aid First-Strand cDNA Synthesis Kit (K1622; Thermo Scientific, United States). The cDNA of each sample was used for RT-PCR with SYBR Green Supermix Kit (Bio-Rad, United States), and the primers used are listed in Table 1. The PCR cycling was performed at 95°C for 3 min, followed by 40 cycles of cycling at 95°C for 10 s, 59.8°C for 30 s, and 55°C for 5 s. Expression of β-actin was used to normalize the differences in total mRNA expression in each sample. The relative expression of each gene was calculated by 2^−△△Ct^_._

**Table 1:** The primer sequences used for real-time PCR.

Gene	Forward primer sequence (5′ → 3′)	Reverse primer sequence (5′ → 3′)
β-actin(pigs)	GGACTTCGAGCAGGAGATGG	AGGAAGGAGGGCTGGAAGAG
IL-1α(pigs)	AGAATCTCAGAAACCCGACTGTTT	TTCAGCAACACGGGTTCGT
IL-1β(pigs)	GCCCTGTACCCCAACTGGTA	CCAGGAAGACGGGCTTTTG
IL-6(pigs)	ATTAAGTACATCCTCGGCAAA	GTTTTCTGCCAGTACCTCC
c-Fos(pigs)	TGCAGACTGAGATCGCCAACC	CCACTCAGATCAAGGGAAGCCACA
c-Myc(pigs)	GAAACAGATCAGCAACAACCGAAA	CATTGTGTGTCCGCCTCTTGTCA
c-Jun(pigs)	CGAGCGCCTGATAATCCAGTCCA	AGCCCTCCTGCTCGTCAGTCAC
STAT1(pigs)	TATAAAGTCATGGCCGCTGA	GTTCCTTTAGGGCCGTCA
Bax(pigs)	GTT TCA TCC AGG ATC GAG CA	TGCAGCTCCATGTTACTGTCC
Bcl-2(pigs)	CTGCACCTGACTCCCTTCACC	TCCCGGTTGACGCTCTCCACA
Bcl-xl(pigs)	GCCACTTACCTGAATGACCA	ATTGTTTCCGTAGAGTTCCAC
US3(pigs)	GGGCTTTCCTGATTTACAAGA TGT	AAGGGCGGCGGACG
β-actin(mouse)	GGCTGTATTCCCCTCCATCG	CCAGTTGGTAACAATGCCATGT
IL-1α(mouse)	AGTTGCCAGAAACACC	GTGGCAATAAACAGCTCT
IL-1β(mouse)	CACTACAGGCTCCGAGA	GCCACAGGTATTTTGTCGTT
IL-6(mouse)	GTCGGAGGCTTAATTACACA	CAAGTGCATCATCGTTGT
MCP-1(mouse)	AAAACCTGGATCGGAAC	TAGCTTCAGATTTACGGGTC
GCSF(mouse)	TCTGCCTCTACCAAGGTCT	CCCCTAGGTTTTCCATCTGC
Bax(mouse)	TTTCATCCAGGATCGAGCAG	CACGTCAGCAATCATCCTC
Bcl-2(mouse)	ACCTGACGCCCTTCACC	CATCTCCCTGTTGACGCTCT
Bcl-xl(mouse)	ATCCCAGCTTCACATAACCC	ATCCGACTCACCAATACCTG

### Animals and experimental design

The animal experimental protocol was approved by the National Institute of Animal Health Animal Care and Use Committee at Sichuan Agricultural University (approval number 2018–012).

Thirty female specific pathogen-free KM mice (body weight 20 ± 2 g) were commercially obtained from the Chengdu Dossy Experimental Animals Co., Ltd. (Chengdu, China), and kept in the BSL-2 lab at Sichuan Agricultural University (Ya’an, China). They were housed at 20°C–25°C with a relative humidity of 55% ± 5% and a 12 h light–dark cycle. After acclimating for a week, the mice were randomly divided into the following three groups (*n* = 10): uninfected-untreated group, infected-untreated group, and treatment group (Myr, 100 mg/kg, dissolved in 0.5% carboxy methyl cellulose sodium solution). The mice, except those in the uninfected-untreated group (0.1 ml PBS), were intraperitoneally injected with 0.1 ml of 2 × 10^4^ TCID_50_ PRV. The mice in the treated groups were orally administered with 0.2 ml myricetin at 1 h post infection, twice daily. In the infected-untreated group and uninfected-untreated group, the mice received 0.2 ml 0.5% CMC-Na. Then, all animals were euthanized by cervical dislocation and subjected to full dissection after the infected-untreated group reached a mortality rate of over 50%.

### Survival rate

The number of deaths in each group was recorded daily. The survival rate was calculated as follows:

Survival rate = number of surviving mice/total number of mice.

### Organ coefficient

After dissection, the heart, liver, spleen, lung, kidney, thymus, and brain were exercised and weighed. The relative organ weight was calculated according to the formula:

Organ coefficient (mg/g) = organ weight/body weight.

### Viral load assay

The heart, liver, spleen, lung, kidney, and brain were collected from each group and immediately frozen with liquid nitrogen, followed by homogenization. Total DNA of tissue sample (20 mg) was extracted by using DNAiso Reagent (D305; TaKaRa, China) according to the manufacturer’s instructions. The viral copies were determined by the fluorescent quantitative polymerase chain reaction (FQ-PCR) method described by Zhao et al. (2017).

### Histopathological examination

During dissection, the liver, spleen, lung, kidney, and brain were taken and fixed in 4.0% paraformaldehyde for 2 weeks, followed by embedding in paraffin. Sections (4 μm) were cut and stained with hematoxylin–eosin (HE) solution. Histopathological changes were observed under a microscope (Nikon Eclipse 80i, Tokyo, Japan). Three slides from different parts of each tissue type (3 mice per group) were analyzed.

### Statistical analysis

All data were representative of at least three independent experiments. Data were presented as means ± standard deviations (S.D.) and analyzed using SPSS 22.0 Statistical Software (IBM, NY, United States). Statistical significance of the data was compared by one-way analysis of variance (ANOVA), *p*-values < 0.05 were considered significant. The bar graph and line chart were prepared by GraphPad Prism 7 (GraphPad Software).

## Results

### Cytotoxicity and antiviral activity of myricetin

**Figure 1:**
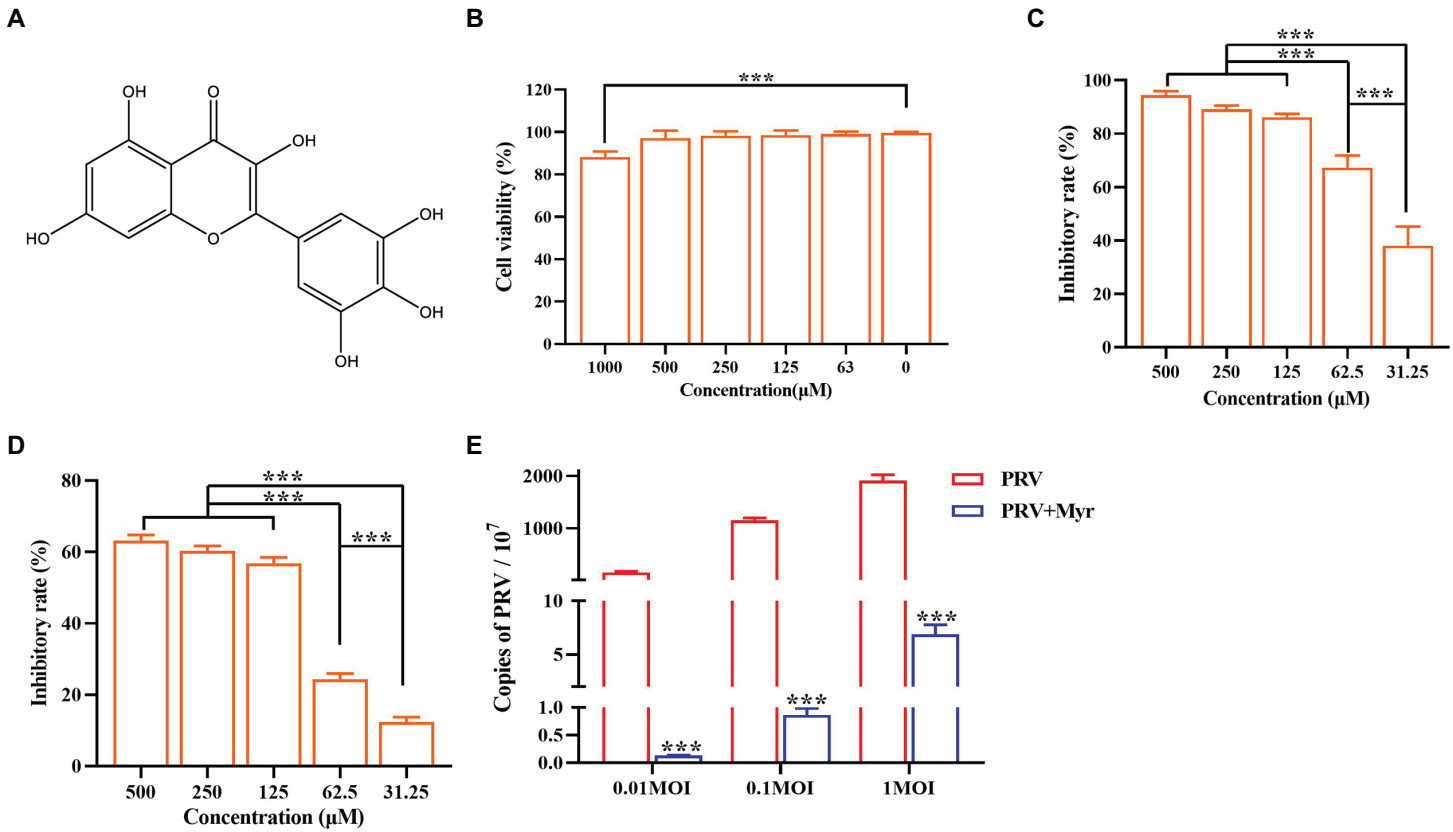
Anti-PRV activity of Myricetin. **(A)** The structural formula of myricetin. **(B)** The cell toxicity of myricetin. PK-15 cells were incubated with different concentrations of myricetin for 48 h, then the cell viability was measured by CCK-8. **(C)** The inhibitory rate of myricetin against PRV. PK-15 cells were infected with PRV (100TCID_50_) and treated with myricetin at the same time. After adsorption for 1 h, the mixture was replaced with fresh medium containing myricetin to culture for 48 h. Then a CCK-8 assay was performed. **(D)** The inhibitory rate of myricetin on PRV replication. PK-15 cells were infected with PRV (100TCID_50_) and treated with myricetin after 1 h post infection. After incubation for 48 h, a CCK-8 assay was used for detection. **(E)** MOI assay. PK-15 cells were infected with PRV (MOI = 0.01, 0.1, and 1, respectively) in the presence of myricetin; after incubated for 1 h at 37°C, the mixture was replaced with fresh compound-containing medium. The DNA copy of PRV was evaluated by FQ-PCR. Values are presented as means ± SD (*n* = 6). PRV, the infected group without treatment; PRV + Myr, the infected group with myricetin treatment. Symbol “^***^” represents *p* < 0.001 between the PRV group and PRV + Myr group.

Myricetin is a polyhydroxyflavone with multiple pharmacological effects and the chemical structure is in Figure 1A. Throughout the tests, the concentration of DMSO in the culture medium is below 0.5%, which could not affect cell growth and virus proliferation. The toxicity study showed that the viability of cells was reduced by 11.9% at a concentration of 1,000 μM (Figure 1B). There was no significant cytotoxicity observed when the concentration is no more than 500 μM, thus the maximum non-toxic concentration of myricetin to PK-15 cells was 500 μM. Myricetin could dose-dependently inhibit PRV growth, and PRV-induced cell deaths were reduced by 96.4% after treatment with myricetin (500 μM; Figure 1C). The IC_50_ was calculated as 42.69 μM (Table 2). Then, the initial infective dose was increased to 0.01, 0.1, and 1 MOI, and myricetin could still significantly inhibit PRV replication in cells at the concentration of 500 μM (Figure 1E). For further confirming the anti-PRV activity of myricetin under post infection, the cells were infected by PRV prior to addition of myricetin. The results showed (Figure 1D) that the anti-PRV potency of myricetin was reduced and the inhibitory rate was 63.2% when the concentration of myricetin was 500 μM.

**Table 2:** Antiviral activity of myricetin against PRV.

Compound	CC_50_ (μM)^a^	IC_50_ (μM)^b^	SI^c^
Myricetin	>1,000	42.69 ± 0.53	>23.24 ± 0.49

a Cytotoxic concentration 50% (CC_50_), the concentration of myricetin induces cell death by 50%.

b Inhibition concentration 50% (IC_50_), the concentration of myricetin reduces PRV-induced cell death by 50%.

c The selectivity index is defined as the ratio of CC_50_ to IC_50_.

**Figure 2:**
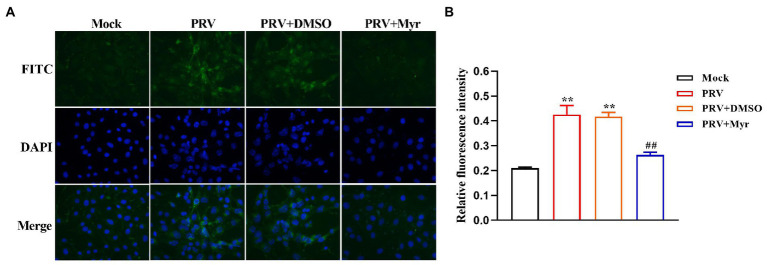
Indirect immunofluorescence. **(A)** PK-15 cells were infected with PRV (MOI = 0.1) in the presence of myricetin, and subjected to immunostaining at 24 h post-infection. Green, PRV virion; blue, nuclei. Magnification, 400X. **(B)** Relative fluoruminosity was counted by using the ImageJ software. The value is defined as the ratio of green fluorescence to blue fluorescence. Mock, the uninfected-untreated group; PRV, the infected group without treatment; PRV + DMSO, the infected group with DMSO treatment (0.5%); PRV + Myr, the infected group with myricetin treatment. Data are presented as mean ± SD, *n* = 6. Symbol “**” represents *p* < 0.01 between the mock group and other groups. Symbol “##” represents *p* < 0.01 between the PRV group and PRV + Myr group.

The propagation of virions in cells was intuitively observed by indirect immunofluorescence. The cells were infected with PRV and treated with 500 μM myricetin at the same time. After infection for 24 h, cells were detected by immunofluorescence assay. The results (Figure 2) showed that cells infected with PRV had a larger nuclear volume and altered morphogenesis, and high fluorescence of viral antigens was detected in PRV-infected cells. In contrast, no nuclear morphology or size was significantly changed and very low fluorescence of viral antigens was detected after myricetin treatment.

### Mode of action

**Figure 3:**
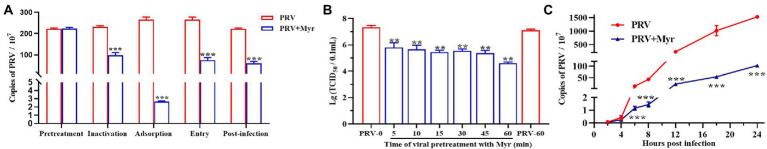
The antiviral mechanism of Myricetin. **(A)** Mode of action study. Myricetin was added on PK-15 cells for 1 h, and after washing, the cells were infected with PRV (Pretreatment). Myricetin was incubated with PRV for 1 h, and then added into cells after dilution (Inactivation). Myricetin was mixed with PRV to infect cells and replaced by new culture medium after adsorbed 1 h at 4°C (Adsorption). Myricetin was added to cells after PRV adsorption for 1 h at 4°C, and then cells were incubated for 1 h at 37°C followed by wahsing with citric acid-sodium citrate buffer (Entry). Myricetin was added after cells was infected with PRV for 1 h at 37°C (Post-infection). **(B)** Time course of virucidal activity. PRV and myricetin were incubated for different time periods and then serially diluted on PK-15 cells for determining viral titers (TCID_50_). **(C)** Growth curve. After infection for 1 h, PK-15 cells were treated with myricetin and the viral gene copies at different time were detected. PRV, the infected group without treatment; PRV + Myr, the infected group with myricetin treatment (0.5 mM). Values are presented as means ± SD (*n* = 6). Symbols “** and ***” represent *p* < 0.01 and *p* < 0.001, respectively, between the PRV group and PRV + Myr group.

To explore the mode of action, several tests based on viral replication cycle were performed. The results (Figure 3A) found that myricetin at the concentration of 500 μM showed a direct virus-killing effect, and also had significant inhibitory effects on the stages of viral adsorption, penetration, and replication. Pretreatment did not show any protective effect on cells (Figure 3A). To further explore the direct viricidal effect of myricetin, we performed a time course of virucidal activity assay and found that coincubation of myricetin with virus at 37°C for 5 min could significantly reduce viral virulence in a time-dependent manner within 60 min (Figure 3B). It is speculated that myricetin may also inactivate progeny viruses and inhibit cell-to-cell transmission. The viral growth curve in the presence of myricetin suggests that it did not significantly inhibit viral replication during the first 4 hpi, but the viral growth significantly inhibited from 4 to 24 hpi (Figure 3C). Taken together, these results suggested that the antiviral effect of myricetin against PRV attributed to direct inactivation and inhibition of viral adsorption and penetration.

### The inhibition of virus-induced apoptosis by myricetin

**Figure 4:**
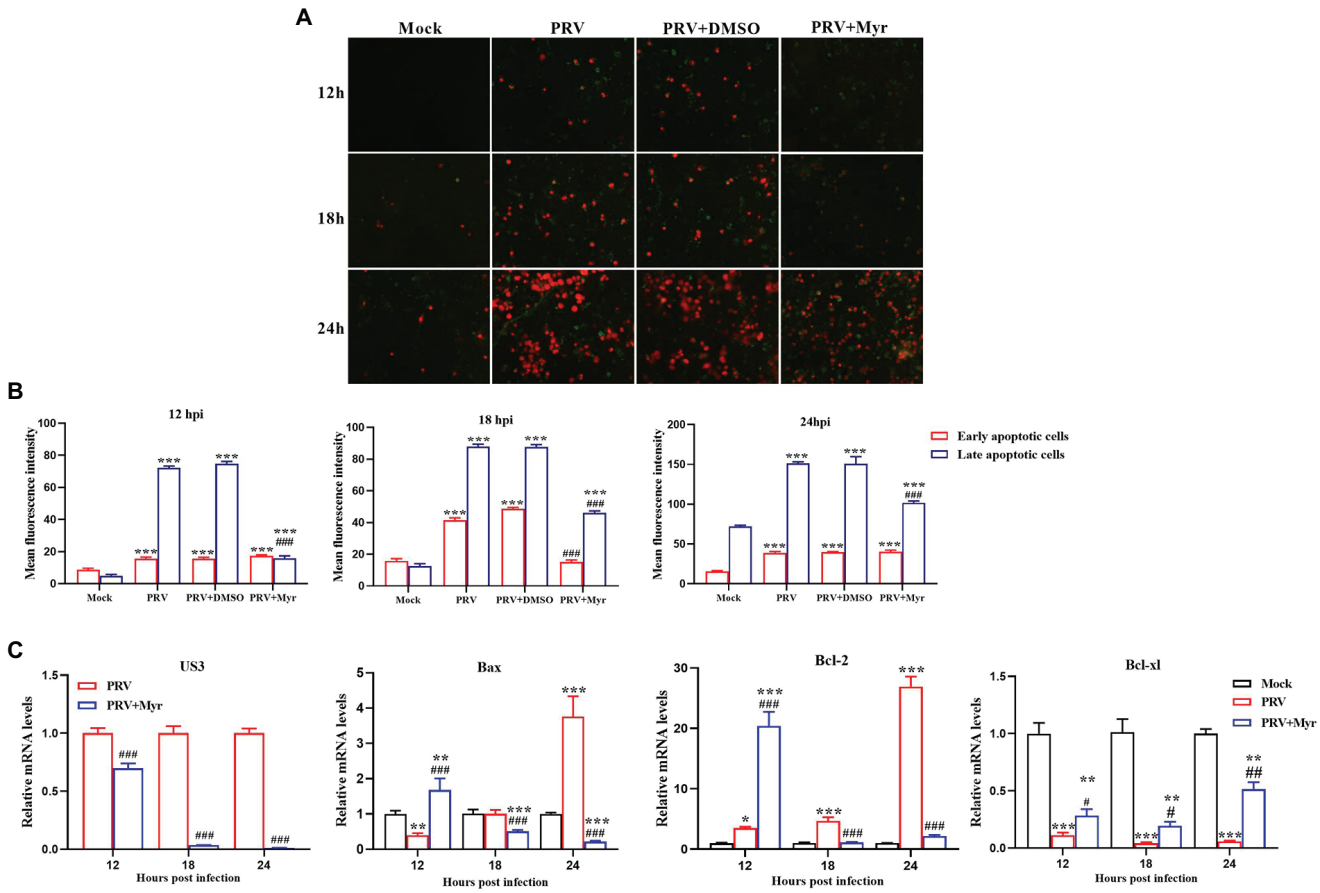
Apoptosis study. **(A)** Myricetin was added after the cells were infected with PRV (MOI = 0.1) for 1 h and subjected to Annexin V-FITC/PI double staining at 12, 18, and 24 hpi, respectively. Green, early period of apoptosis; Red, later period of apoptosis or necrosis. Magnification, 200X. **(B)** Relative fluorescence intensity was counted by using the ImageJ software. **(C)** Myricetin was added after the cells infected with PRV (MOI = 0.1) for 1 h and Total RNA was extracted by TRIzol reagent at 12, 18, and 24 hpi, respectively. Then, the total RNA was reverse-transcribed by Revert Aid First-Strand cDNA Synthesis Kit. Expression of each gene was determined by real-time PCR. Mock, the uninfected-untreated cells; PRV, the infected group without treatment; PRV + DMSO, the infected group with DMSO treatment (0.5%); PRV + Myr, the infected group with myricetin treatment (0.5 mM). Values are presented as means ± SD (*n* = 6). Symbols “*, **, and ***” represent *p* < 0.05, *p* < 0.01, and *p* < 0.001, respectively, between the mock group and other groups. Symbols “#, ##, and ###” represent *p* < 0.05, *p* < 0.01, and *p* < 0.001, respectively, between the PRV group and other groups.

Viral infection often causes apoptosis and necrosis (Cheung et al., 2000). Annexin V-FITC/PI double staining was employed to explore the inhibitory effect of myricetin on PRV-causing apoptosis. The results (Figure 4A) showed that apoptosis occurred in the uninfected cells after 18hpi. In the infected cells, apoptosis was detected at 12 hpi and most of the cells were shown in late apoptosis or necrosis at 24 hpi. After myricetin treatment (500 μM), cells showed a reduced degree of apoptosis when compared to untreated cells and the relative fluorescence intensity what’s we counted were consistent with the above results (Figure 4B). To further explore the effect of myricetin on the apoptosis caused by PRV infection, we examined the expression of apoptosis-related genes. The results (Figure 4C) found that PRV-infected cells could significantly up-regulate the expression of antiapoptotic gene Bcl-2 at 12, 18, and 24 hpi, but the pro-apoptotic gene Bax was significantly downregulated at 12 hpi until its expression was significantly upregulated at 24 hpi. However, the antiapoptotic gene Bcl-xl was significantly downregulated at 12, 18, and 24 hpi. After 0.5 mM myricetin treatment, the regulation of PRV infection on apoptosis-related cytokine expression was reversed. Not only that, the expression of PRV apoptosis-related gene US3 gene was suppressed in the PRV after myricetin treatment, and the degree of inhibition was positively correlated with the time of drug action. The above showed that myricetin can inhibit apoptosis by inhibiting PRV apoptosis-related gene and regulating apoptosis-related cytokines.

### The changes of NF-κB and P38 MAPK signaling pathways

**Figure 5:**
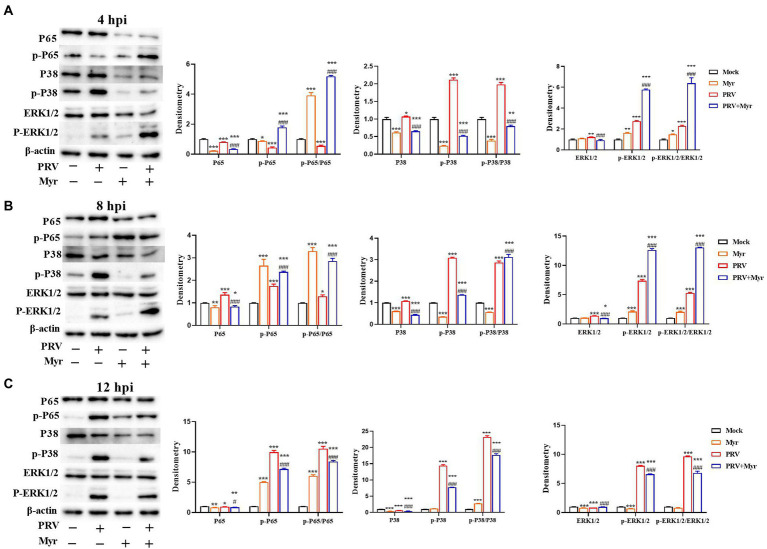
The changes of the NF-κB and MAPK pathways. The PK-15 cells were infected with or without PRV (MOI = 1) in the presence or absence of myricetin. Expressions of P65, p-P65, ERK1/2, p-ERK1/2, P38, and p-P38 were determined by Western blotting at 4 **(A)**, 8 **(B)**, and 12 **(C)** hpi. Mock, the uninfected-untreated cells; Myr, the uninfected group with myricetin treatment. PRV, the infected group without treatment; PRV + Myr, the infected group with myricetin treatment. Data are presented as mean ± SD, *n* = 6. Symbols “*, **, and ***” represent *p* < 0.05, *p* < 0.01, and *p* < 0.001, respectively, between the mock group and other groups. Symbols “# and ###” represent *p* < 0.05 and *p* < 0.001, respectively, between the PRV group and PRV + Myr group.

Viral infection usually causes changes in cellular signaling pathways to facilitate their own proliferation (Sun et al., 2017). Studies have found that the MAPK and NF-κB pathways are associated with viral infection (Xu et al., 2019). The changes of NF-κB and MAPK signal pathways were shown in Figure 5. After 4 h of viral infection (Figure 5A), the expressions of P65, p-P65 as well as p-P65/P65 were significantly reduced in the PRV-infected cells compared to uninfected control; the expressions of ERK1/2, p-ERK1/2, P38, and p-P38 as well as p-ERK1/2/ERK1/2 and p-P38/P38 were elevated when compared with uninfected control. After treated with myricetin, the expressions of p-P65 and p-ERK1/2 as well as p-P65/P65 and p-ERK1/2/ERK1/2 were significantly enhanced and the expressions of ERK1/2, P38, p-P38 as well as p-P38/P38 were significantly reduced when compared to the infected-untreated group. At 8 hpi (Figure 5B), the levels of P65, p-P65, p-P65/P65, ERK1/2, p-ERK1/2, P38, p-P38, p-ERK1/2/ERK1/2, and p-P38/P38 were significantly increased in the PRV-infected cells when compared with uninfected control. In contrast, the levels of p-P65, p-P65/P65, p-ERK1/2 as well as p-ERK1/2/ERK1/2 were significantly elevated, and the levels of ERK1/2, P38, p-P38 as well as p-P38/P38 was significantly reduced after myricetin treatment. At 12 hpi (Figure 5C), compared with the uninfected control, PRV infection increased the levels of p-P65 and p-P65/P65; the expressions of ERK1/2 and P38 were significantly reduced, and the expressions of p-ERK1/2 and p-P38 as well as p-ERK1/2/ERK1/2 and p-P38/P38 were significantly elevated. After myricetin treatment, the levels of P65, p-P65, p-P65/P65, p-ERK1/2, P38, p-P38, p-ERK1/2/ERK1/2, and p-P38/P38 were significantly reduced, whereas the expression of ERK1/2 was significantly increased when compared with the PRV-infected cells. These results suggested that PRV infection could suppress the NF-κB pathway at 4 hpi, then turned into activation at 8 and 12 hpi. Myricetin treatment can activate the NF-κB pathway at 4 and 8 hpi, then turned to inhibition at 12 hpi. It is also suggested that PRV infection could activate the MAPK pathway, and myricetin could inhibit the persistent activation.

### The changes of gene expressions

**Figure 6:**
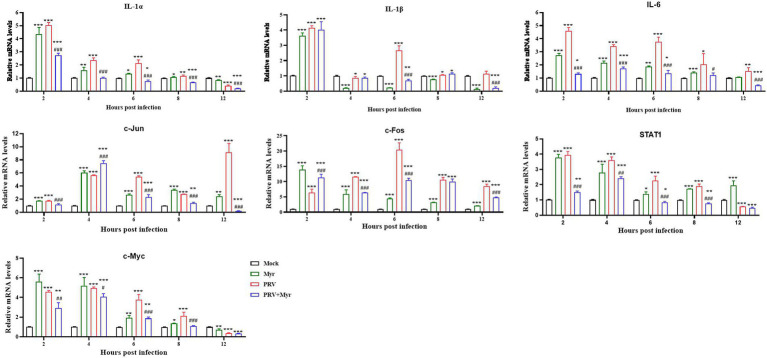
Gene expressions. The PK-15 cells were infected with or without PRV (MOI = 1) in the presence or absence of myricetin. The total mRNA of each sample was extracted at 2, 4, 6, 8, and 12 hpi, respectively, and subjected to real-time PCR to test the expressions of the target genes of NF-κB (IL-1α, IL-1β, and IL-6) and MAPKs (c-Jun, STAT1, c-Fos, and c-Myc) signaling pathways. Mock, the uninfected-untreated cells; Myr, the uninfected group with myricetin treatment; PRV, the infected group without treatment; PRV + Myr, the infected group with myricetin treatment. Data are presented as mean ± SD, *n* = 6. Symbols “*, **, and ***” represent *p* < 0.05, *p* < 0.01, and *p* < 0.001, respectively, between the mock group and other groups. Symbols “#, ##, and ###” represent *p* < 0.05, *p* < 0.01, and *p* < 0.001, respectively, between the PRV group and PRV + Myr group.

Previous studies found that PRV infection can affect NF-κB and MAPK pathways, and the effect of myricetin on relevant target gene expressions was tested. The results are shown in Figure 6. The IL-1α, IL-1β, and IL-6 are associated with the NF-κB pathway, the relative expressions of IL-1α and IL-6 were significantly increased at 2, 4, 6, and 8 hpi in comparison with the uninfected-untreated control, which were significantly decreased by myricetin treatment. The expression of IL-1β was increased by PRV infection at 2, 6, 8, and 12 hpi, but decreased at 4 hpi, whereas it was inhibited at 6 and 12 hpi after myricetin treatment. The c-Jun, STAT1, c-Fos, and c-Myc are associated with the MAPK pathway. The expressions of c-Jun and c-Fos were upregulated within 12 hpi in comparison with the uninfected-untreated control. The expressions of STAT1 and c-Myc were upregulated within 10 hpi, but decreased at 12 hpi. The changes in these gene expressions caused by PRV infection are inhibited by myricetin.

### The antiviral activity of myricetin in PRV-infected mice

**Figure 7:**
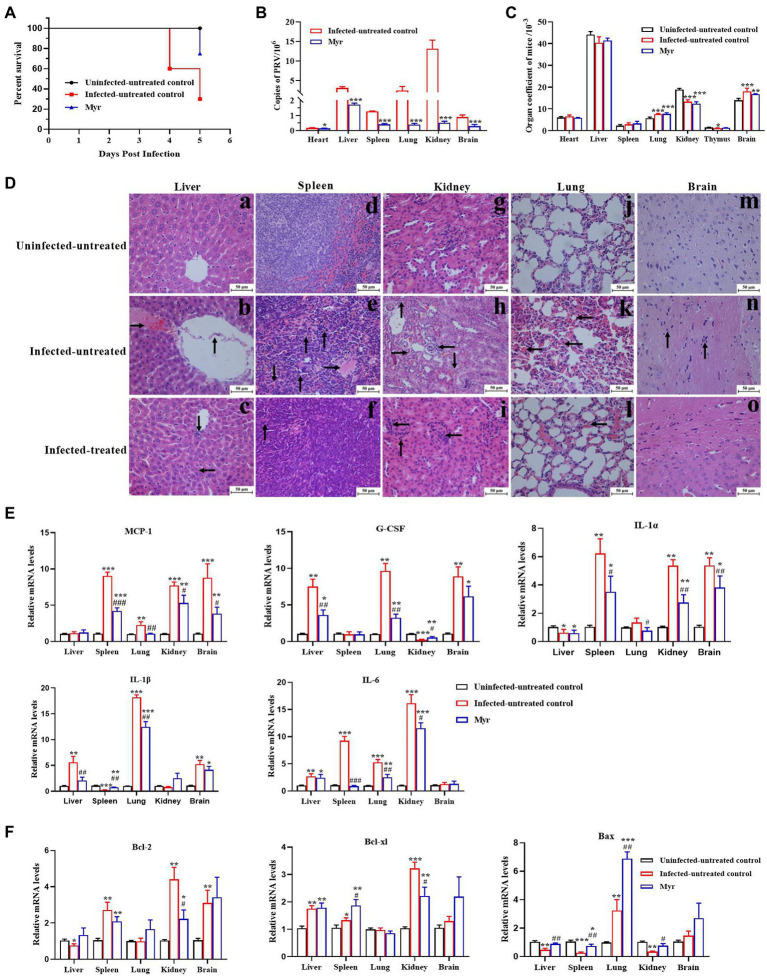
Antiviral activity of myricetin in PRV-infected mice. **(A)** The survival rate of mice in each group depicted by Kaplan–Meier survival plots. The survival rate = number of surviving mice/total number of mice. **(B)** Virus load of liver, spleen, lung, kidney, and brain in each group (*n* = 3). The organs were frozen with liquid nitrogen, followed by homogenization. Total DNA of tissue sample was extracted and viral copies were determined by FQ-PCR. **(C)** Organ coefficient of mice. The heart, liver, spleen, lung, kidney, thymus, and brain were collected and weighed after dissected. The organ coefficient (mg/g) = organ weight/body weight. **(D)** Histopathological examination. In the liver **(a–c)**, PRV infection caused disordered arrangement of hepatic cords, shedding of blood vessel walls (denoted by arrowhead “↑”), and forming a clot (denoted by arrowhead “→”); The main changes were clustering of lymphocytes (denoted by arrowhead “↓”) and necrosis of liver cells (denoted by arrowhead “←”) after myricetin treatment. In the spleen **(d–f)**, PRV infection formed a clot (denoted by arrowhead “→”), bleeding (denoted by arrowhead “↓”) and hemosiderosis (denoted by arrowhead “↑”); after myricetin treatment, the main changes were hemosiderosis (denoted by arrowhead “↑”). In the kidney **(g–i)**, PRV infection caused cell granule denaturation (denoted by arrowhead “↑”), hyperemia (denoted by arrowhead “→”), hematolysis (denoted by arrowhead “↓”) and clustering of lymphocytes (denoted by arrowhead “←”); after myricetin treatment, the main change was clustering of lymphocytes (denoted by arrowhead “←”). In the lung **(j–l)**, PRV infection caused blood congestion and blood stasis in the alveolar septal capillaries (denoted by arrowhead “←”); after myricetin treatment, alveolar congestion and blood stasis were alleviated. In the brain **(m–o)**, PRV infection caused the glial cells surrounding the neurons (denoted by arrowhead “↑”); no significant changes were detected after myricetin treatment. **(E,F)** Expression levels of inflammatory factors **(E)** and apoptotic factors **(F)**. Total RNA of different organs was extracted and reverse-transcribed for RT-PCR assay. All data are presented as mean ± SD, *n* = 3. Symbols “*, **, and ***” represent *p* < 0.05, *p* < 0.01, and *p* < 0.001, respectively, between the uninfected-untreated group and other groups. Symbols “#, ##, and ###” represent *p* < 0.05, *p* < 0.01, and *p* < 0.001, respectively, between the infected-untreated group and infected-treated group.

To further confirm the anti-PRV activity *in vivo*, the protective effects of myricetin on PRV-infected mice were tested. The results were shown in Figure 7. The mice began dying at 4 days post infection (dpi) with a mortality rate of 40%, and mortality rate as high as 70% at 5 dpi. After myricetin treatment, the mice began dying at 5 dpi with a mortality rate of 30% (Figure 7A). These results suggested that myricetin could prolong the survival of infected mice and enhance the survival rate.

The PRV proliferation was detected in each tissue by FQ-PCR. As shown in Figure 7B, PRV is replicated the most in the kidney, second by the liver, lung, spleen, brain, and the least proliferation in the heart. After myricetin treatment, the copy number of PRV in each organ was greatly reduced, indicating that myricetin can effectively inhibit the virus proliferation.

Organ index is a commonly used index in the toxicology experiment, which can reflect whether the body is in a pathological state. Our results showed that (Figure 7C) the organ indices of liver, kidney and thymus were significantly increased in PRV-infected mice, but lung and brain indices were significantly reduced. The results indicate that the liver, kidney, and thymus of infected mice may appear some degenerative changes, and lung and brain may appear edema, congestion, and other changes. After myricetin treatment, there was a certain degree of recovery in the organ indices of liver, thymus, and brain. Through histopathological examination, all the tested organs had different levels of pathological damages after infection with PRV (Figure 7D). In the liver, PRV infection caused disordered arrangement of hepatic cords, shedding of blood vessel walls, and forming a clot. The main changes were clustering of lymphocytes and necrosis of liver cells after myricetin treatment. In the spleen, PRV infection caused forming a clot, bleeding, and hemosiderosis. After myricetin treatment, the lesion was only hemosiderosis. In the kidney, PRV infection caused cell granule denaturation, hyperemia, hematolysis, and probable clustering of lymphocytes. After myricetin treatment, the main change was clustering of lymphocytes. In the lung, PRV infection caused blood congestion and blood stasis in the alveolar septal capillaries. Myricetin treatment reduced alveolar congestion and blood stasis. In the brain, PRV infection caused the glial cells surrounding the neurons, and there was no significant pathological damage in the brain after myricetin treatment.

The gene expression levels of inflammatory cytokines and apoptotic factors in lung, kidney, brain, spleen, and liver after PRV infection were determined by RT-PCR (Figures 7E,F). The expressions of IL-1α and Bax in liver were suppressed after PRV infection, while the G-CSF, IL-1β, IL-6, and Bcl-xl were upregulated. The expression of MCP-1, IL-1α, IL-6, Bcl-xl, and Bcl-2 was increased in the spleen, while the expression of IL-1β and Bax was downregulated. In the lung, all genes were upregulated, except for no significant change in the expression levels of Bcl-xl, and Bcl-2. Expressions of MCP-1, IL-1α, IL-6, Bcl-xl, and Bcl-2 were all increased in the skidney, whereas the expression levels of G-CSF and Bax were decreased. The expression levels of MCP-1, G-CSF, IL-1α, IL-1β, Bcl-xl, and Bcl-2, as well as Bax were also upregulated in the brain. The expression levels of inflammatory factors and apoptosis factors were recovered in each organ after myricetin treatment.

## Discussion

Myricetin is a polyhydroxy dietary flavonoid compound widely found in fruits, vegetables, and tea with antiviral activity against some viruses, such as HSV, HIV, SARS-CoV (Ono et al., 2010; Yu et al., 2012; Pasetto et al., 2014; Ren et al., 2018; Li et al., 2020). Herpesvirus hosts have a wide range and still cause serious harm to humans and livestock and poultry today. Myricetin can target the viral gD protein and downregulate the cellular EGFR/PI3K/Akt pathway to inhibit HSV infection and replication. PRV belongs to a member of the family *Herpesviridae* and is often regarded as a model virus to study herpesviruses (Karlin et al., 1994). In this study, myricetin can achieve antiviral effects by directly inactivating the virus and inhibiting viral adsorption and penetration. Myricetin can also inhibit PRV-induced apoptosis and regulate the body immune and inflammatory responses caused by viral infection through the NF-κB and MAPK signal pathways. Thus, myricetin exhibited the potential to developing as an antiviral drug against herpesviruses.

The proliferative process of most viruses includes adsorption, penetration, replication, and release, and blocking any stage may inhibit viral infection. The approved anti-herpesvirus drugs are mainly a class of nucleoside analogs that selectively inhibit viral DNA polymerases, such as acyclovir, valacyclovir, and penciclovir. These drugs have adverse reactions involving the kidney, nerve, and heart in different ways of administration (Arndt, 1988; Haefeli et al., 1993; Izzedine et al., 2005; Ono et al., 2010; Gill et al., 2017). And long-term use has led to the production of resistant strains, making the treatment of herpes virus infections more difficult (Kukhanova et al., 2014). In this study, myricetin has a different antiviral mechanism from approved drugs. Pre-incubation of PRV with myricetin can reduce its infective ability and also inhibit PRV adsorption, penetration, and replication phases. It has been shown that PRV progeny viruses are usually detected upon infection with 4–5 h in commonly used cell lines (Pomeranz et al., 2005). Our results suggested that inhibitory effect of myricetin after PRV entry into cells may be due to its direct inactivation and inhibiting adsorption and penetration to suppress viral transmission from cell to cell, but it does not rule out that myricetin has an effect of inhibiting PRV replication. The initial attachment of herpesviruses to cells was achieved by binding of the virus gC glycoproteins to the HSPG and CSPG receptors on the cell membrane. Although gC glycoproteins are important for viral adhesion cells, this effect can be replaced by gB when lacking (Wudunn and Spear, 1989; Herold et al., 1991; Cheshenko and Herold, 2002). In addition, the viral envelope proteins, gD, gH, and gL, are also taking part in viral entry, release, and cell-to-cell transmission, and these glycoproteins are also strongly associated with host immune defense (Mettenleiter, 1996, 2000). Myricetin may bind to glycoproteins on the herpes virus membrane to block PRV entry. Other flavonoids were found to exhibit similar antiviral activity (Ninfali et al., 2020).

Stimulation to cells caused by herpes virus infection often triggers autophagy and apoptosis, which is detrimental to viral proliferation. Therefore, most viruses in the early stage of infection can promote the production of antiapoptotic factors to delay cell death to obtain maximum viral production, and in the later stages of infection, virus will promote apoptosis to release the progeny virus (Teodoro and Branton, 1997; Roulston et al., 1999). Annexin V-FITC/PI double staining is a commonly used method to detect apoptosis (Hu et al., 2019). In this study, we found that only very few cells developed apoptosis early in PRV infection, whereas after 18 h of infection, large number of cells gradually developed apoptosis and necrosis. Apoptosis induced by viral infection was controlled after myricetin treatment. Not only that, by examining the expression of apoptosis-related cytokines as well as PRV US3 genes, it was found that myricetin can significantly inhibit the regulation of PRV infection on Bcl-2, Bcl-xl, and Bax and also inhibit US3 gene expression in PRV. It was found that in the early stages of α-herpesvirus infection, the protein kinase encoding by US3 plays an important role in promoting cell survival to improve self-production during infection of host cells (Deruelle et al., 2010). The US3 protein kinase can inhibit early apoptosis in infected cells by inhibiting the overexpression of relevant apoptotic factors such as Bcl-2 family members, as well as by activating the PI3-K/Akt and NF-κB signaling pathway (Ogg et al., 2004; Chang et al., 2013). US3 also reduces autophagy levels in cells by activating the AKT/mTOR signaling pathway (Sun et al., 2017). However, the inhibitory effect of herpes virus on apoptosis is only temporary. PRV was found to activate caspase-3 expression in neurons during late in infection. In addition, ERK1/2 was also found to be activated to regulate cell apoptosis upon PRV infection (Marcaccini et al., 2006; Schulz et al., 2014). It was found that myricetin had both anti-apoptotic and pro-apoptotic effects. Myricetin can promote apoptosis and autophagy in various cancer cells by regulating the enzymatic activity associated with the development of cancer, as well as signaling pathways (Ci et al., 2018; Li et al., 2019; Xie et al., 2019). In addition, myricetin inhibits high glucose and oxidative stress-induced apoptosis through the inhibition of CDK-5 activity and by regulating apoptosis-related proteins (Karunakaran et al., 2019; Aminzadeh and Bashiri, 2020). Myricetin also protects cells from H_2_O_2_-induced apoptosis by regulating PI3K/Akt and MAPK signaling pathways (Kang et al., 2010). Thus, inhibition of PRV-induced apoptosis by myricetin may act by inhibiting the gene expression of PRV US3 and regulating apoptosis-related signaling pathways.

Myricetin can regulate several signaling pathways, such as MAPK, PI3K/AKT/mTOR, and IκB/NF-κB, leading to enhancement of immunomodulatory function and inhibition of cytokine storms (Cheung et al., 2000). PRV infection leads to an uncontrollable systemic inflammatory response in the body, which is greatly related to the continuous activation of the NF-κB pathway (Romero et al., 2020). The MAPK signaling pathway plays an important role in regulating cell growth, differentiation, inflammation, apoptosis, and other stress responses (Chong et al., 2003). The MAPK and NF-κB pathways were found to be associated with viral infection (Sun et al., 2017; Xu et al., 2019). When virus invades cells, the cells will trigger a series of cellular immune responses to resist the virus invasion and the virus also produces an immune escape mechanism (Banks and Rouse, 1992). In this study, the NF-κB and MAPK signaling pathways that are closely related to viral infection were detected. NF-κB pathway dysregulation is closely linked with allergies, autoimmune disorders among other pathologies (Zhang et al., 2017). Thus, aberrant PRV-induced NF-κB activation may therefore serve as a viral immune evasion strategy. HSV-1 inhibits the NF-κB pathway by binding to IκBα through its early protein ICP 27, enabling it to escape the immune response of the host cells early in infection (Jin et al., 2008). Similarly, it was found that PRV degraded P65 through its UL24 protein to inhibit the NF-κB pathway for immune escape (Wang et al., 2020). Our results suggested that myricetin inhibited PRV infection in the early period of infection through the activation of the NF-κB pathway and regulating the immune response. However, PRV does not exert a sustained inhibition effect on the NF-κB pathway. It was found that PRV caused persistent NF-κB activation when infecting epithelial cells, thus modulating the host pro-inflammatory response for immune escape purposes, and that activation suppresses apoptosis (Romero et al., 2020). Myricetin can alleviate the inflammatory response by inhibiting the activation of PRV-induced NF-κB in the later stages of infection. Alternatively, PRV can perform immune escape by modulating the cellular antiviral immune response and inflammatory responses mediated by the JAK/STAT, MAPK, and NLRP3 pathways (Li et al., 2022). An ethyl acetate fraction of flavonoids from *Polygonum hydropiper* L. was found to reduce the PRV-induced inflammatory response by reducing the phosphorylation of the MAPK key proteins and the displacement of NF-κB p65 into the nucleus (Ren et al., 2020).The P38 MAPK signaling pathway delivers extracellular signals to the nucleus and plays an important role in regulating multiple cellular processes, such as the cell life cycle and the immune response (Ono and Han, 2000; Cuadrado and Nebreda, 2010). Viral infection activates the P38 MAPK pathway to promote its own replication, and drug blocking activation of P38 effectively suppresses viral infection (Cheng et al., 2020). It was found that the cell apoptosis caused by PRV infection was closely correlated with the activation of the P38 and the JNK/SAPK signaling pathways (Marcaccini et al., 2006). This is consistent with this study that the P38 MAPK pathway is continuously activated after PRV infection and PRV infection promoted apoptosis in a time-dependent manner. However, viral-induced P38 activation and apoptosis were inhibited by myricetin treatment. ERK1/2, also belonging to the MAPK pathway as P38, is an evolutionarily conserved signaling pathway that is closely related to cell proliferation, differentiation, apoptosis and the transcription and expression of certain genes (Chong et al., 2003; O’Neill and Kolch, 2004). We found that PRV infection consistently activates ERK1/2, and after myricetin treatment the activation was promoted at 4 and 8hpi, turning into inhibition at 12 hpi. The gE glycoprotein of PRV was found to induce T lymphocyte migration by inducing the phosphorylation of ERK1/2 in T lymphocytes, thereby spreading the infection to other susceptible cells (Pontes et al., 2014). Alternatively, PRV participates in mitosis and apoptosis to disrupting the nuclear membrane by activating the ERK1/2 pathway, and releasing nucleocapsids from the nucleus to the cytosol, what is a process important in the herpesvirus replication cycle (Schulz et al., 2014). These results suggested that myricetin regulates apoptosis and nuclear membrane rupture by regulating PRV-induced ERK1/2 activation to affecting viral replication.

To further validate the regulatory role of myricetin on the both pathways, we examined the expression of relevant genes in the pathway. IL-1α and IL-1β, the first described members of the IL-1 family, control the inflammatory responses to tissue damage by identifying pathogen molecules and the signaling molecules released from damaged cells. IL-1α activation by NF-κB signaling regulates the release of inflammatory cytokines, including IL-1α, IL-1β, and IL-6 (March et al., 1985; Kelly-Welch et al., 2005; Dinarello, 2009; Lawrence, 2009). PRV infection was found to cause a systemic lethal inflammatory response, increasing the contents of major pro-inflammatory cytokines such as IL-β and IL-6 (Laval et al., 2018; Ren et al., 2021). In previous experiments, we found that PRV infection could continuously activate the NF-κB pathway, and that the activation of this pathway would promote the expressions of related cytokines, such as IL-1α, IL-1β, and IL-6, which was consistent with the results of cytokine expressions as we examined. Thus, it is shown that myricetin modulates pro-inflammatory cytokine production to inhibit the inflammatory responses by inhibiting the PRV-induced NF-κB signaling. Activation of the MAPK pathway facilitates viral replication and is involved in the cellular immune response and apoptosis caused by viral infection. The c-Jun, STAT1, c-Fos and c-Myc are transcription factors in the MAPK signaling pathway and transcription of pro-apoptotic and anti-apoptotic factors including Bax is also regulated by JNK and P38 in MAPK (Green and Llambi, 2015; Kumar et al., 2018). In our study, PRV infection significantly upregulated expression levels of c-Jun, c-Fos, c-Myc, as well as STAT1, while expression levels of these transcription factors were significantly reduced upon myricetin treatment (Figure 6). PRV infection activated MAPK signaling and changed the expression level of c-Jun, c-Fos, c-Myc, and STAT1 which are the downstream target genes in the pathway. Moreover, c-Jun and c-Fos can form a AP-1 heterodimeric complex, implying that AP-1 is also involved in PRV infection and regulates cell apoptosis (Glover and Harrison, 1995). In our study, PRV infection would continuously activate the MAPK pathway, while the activation of the MAPK pathway modulates the expressions of related cytokines, which was consistent with the results of cytokine expressions. These results suggest that myricetin inhibits PRV infection and controls cell apoptosis by regulating the expression levels of relevant transcription factors in the MAPK pathway.

Pseudorabies virus hosts a wide range, and can infect a variety of other mammals in addition to primates (Woźniakowski and Samoreksalamonowicz, 2015). *In vivo* study, PRV-infected animal models (often mice and rabbits) through artificial infection were used. The routes for PRV infection based on the research purpose is different, mainly including nasal drip, abdominal injection, intramuscular injection, foot injection, and subcutaneous injection. No matter the infection route, PRV will lead to unnatural host severe itching (Laval and Enquist, 2020). In order to explore whether myricetin could inhibit PRV infection *in vivo*, we established a mouse model by intraperitoneal injection. The clinical symptoms included manifested as scratching the injection site, decreased diet and mental depression. The mice were orally treated with 100 mg/kg myricetin twice a day because the oral elimination half-life in rats was only 3.53 h (Li et al., 2021). After myricetin treatment, the survival time of the mice was prolonged; the pathological damages caused by infection were also relieved; the proliferation of PRV was also inhibited in each organ (Figures 7A–D). *In vitro* study, myricetin mainly acted on the direct killing of virus and inhibiting the adsorption or penetration, so it is more suitable for the prevention of virus infection or combination with other drugs which acting in the virus replication stage. Studies have found that PRV infection will lead to a series of host histopathological damage. In PRV-infected pigs, the brain appeared congestion mainly non-suppurative encephalitis and the formation of “satellitosis,” “neuronophagy” phenomenon, and the lung mainly appeared pathological changes of congestion and edema (Yang et al., 2016a). After mice infected with PRV, similar pathological changes were exhibited, such as encephalitis and pulmonary hemorrhage edema, even die in a short period of time, which were consistent with to our results (Ren et al., 2021). The rapid death in mice may attribute to pyroptosis and dysregulation of pro-inflammatory factors which induced unbalance of anti-inflammatory and pro-inflammatory cytokines, leading to a “cytokine storm” (Sun et al., 2021). Viral infection usually causes cell apoptosis and necrosis and an inflammatory response (Knipe and Howley, 2013). PRV can mediate cell apoptosis *in vitro* and *in vivo* by regulating a range of signaling pathways as well as the expression of caspase-3 (Marcaccini et al., 2006; Chang et al., 2013; Sun et al., 2017). Herpes simplex virus can also mediate the apoptosis of infected cells by regulating the expression of Bcl-2 family members (Mettenleiter, 2000). In the present study, ocular purulence in infected mice was observed which may be due to conjunctivitis caused by PRV infection, and the increased index of brain and lung organs may be due to edema and congestion. At the same time, through histopathological observation, different degrees of inflammatory responses in the test organs (Figure 7D). The expression levels of the inflammation-related cytokines, MCP-1, G-CSF, IL-1, IL-1, and IL-6, as well as the apoptosis-related cytokines, Bcl-2, Bcl-x, and Bax were altered in organs of the infected mice (Figure 7E). The congenital immune system, the first line of defense against foreign invasion, often acts by initiating the inflammatory response, but the uncontrolled inflammatory response usually leads to organ failure and dysfunction of the body and even death (Tisoncik et al., 2012). In influenza, excessive expressions of pro-inflammatory cytokines also known as “cytokine storm” is the cause of various complications and death (Liu et al., 2016). And the expression levels of interferons, tumor necrosis factors, interleukins or chemotactic factors would significantly increase (Beigel et al., 2005). Similarly, Sindbis virus infection can cause elevated expression levels of interferons, TNF-α, and IL-6 in the serum of newborn mice, thus causing a systemic inflammatory response (Klimstra et al., 1999). Myricetin has a variety of pharmacological activities, including anti-inflammatory effects. It was shown that myricetin can exert a protective effect on LPS-induced early inflammation in cardiomyocytes by inhibiting IL-6 and IL-1β expression through NF-B signaling pathway (Chen and Fan, 2018). Similarly, myricetin also inhibited the apoptosis and inflammatory response of human umbilical vein endothelial cells HUVECs by upregulating the expression of miR-29a-3p (Bai et al., 2021). In general, myricetin can enhance body immunity and inhibit cytokine storms by regulating multiple signaling pathways (Cheung et al., 2000). Based on the above studies, the protective effect of myricetin on PRV-infected mice may be due to its mitigation of cytokine storms by regulating cytokine expressions through related signaling pathways.

## Conclusion

Myricetin can effectively inhibit the infection of herpes virus and prevent viral entry into cells at the early stage of infection. It can regulate the NF-κB and MAPK pathways to inhibit viral replication and control the inflammatory response and apoptosis caused by viral infection. Myricetin also inhibits PRV proliferation in mice and alleviates the inflammatory response induced by infection to prolong the survival of infected mice. The study provided a new strategy for the treatment of herpes virus infection with dietary flavonoids.

## Data availability statement

The original contributions presented in the study are included in the article/supplementary material, further inquiries can be directed to the corresponding authors.

## Ethics statement

The animal study was reviewed and approved by National Institute of Ethics Committee at Sichuan Agricultural University [approval number SYXK (Sichuan) 2018-012].

## Author contributions

HH, ZH, and YZh carried out laboratory work, wrote the manuscript, and performed the data analyses. HH and XS designed the experiment. HW, XL, LL, XZ, LY, GY, YZo, YC, and HT carried out laboratory work. ZY, RJ, HZ, and XS conceived and supervised this work. All authors contributed to the article and approved the submitted version.

## Funding

This research was financially supported by the Natural Science Foundation of Sichuan Province (2022NSFSC1681) and the Program Sichuan Veterinary Medicine and Drug Innovation Group of China Agricultural Research System (SCCXTD-2020-18).

## Conflict of interest

ZH was employed by Shandong New Hope Liuhe Agriculture and Animal Husbandry Technology Co., Ltd. (Dezhou, China).

The remaining authors declare that the research was conducted in the absence of any commercial or financial relationships that could be construed as a potential conflict of interest.

## Publisher’s note

All claims expressed in this article are solely those of the authors and do not necessarily represent those of their affiliated organizations, or those of the publisher, the editors and the reviewers. Any product that may be evaluated in this article, or claim that may be made by its manufacturer, is not guaranteed or endorsed by the publisher.
